# Symptom experience and symptom distress in patients with malignant brain tumor treated with proton therapy: A five-year follow-up study

**DOI:** 10.1016/j.tipsro.2024.100269

**Published:** 2024-08-16

**Authors:** Kristin Kunni, Ulrica Langegård, Emma Ohlsson-Nevo, Ingrid Kristensen, Katarina Sjövall, Per Fessé, Linda Åkeflo, Karin Ahlberg, Per Fransson, on behalf of the Proton Care Study Group

**Affiliations:** a Skandion Clinic, Uppsala, Sweden; b Department of Oncology, Sahlgrenska University Hospital, Gothenburg Sweden; c Institute of Health and Care Sciences, Sahlgrenska Academy, University of Gothenburg, Sweden; d Department of Oncology, Faculty of Medicine and Health, Örebro University, Örebro, Sweden; e University Health Care Research Center, Faculty of Medicine and Health, Örebro University, Örebro, Sweden; f Department of Haematology, Oncology and Radiation Physics, Lund University Hospital, Lund, Sweden; g Department of Clinical Sciences, Oncology and Pathology, Lund University, Lund, Sweden; h Department of Health and Society, Kristianstad University, Kristianstad, Sweden; i Department of Nursing, Umeå University, Umeå, Sweden; j Centre for Research and Development, Uppsala University/Region Gävleborg, Gävle, Sweden

**Keywords:** Symptom experience, Symptom distress, Malignant braintumor, Proton therapy, Radiotherapy, Patient-reported outcome, Quality of life

## Abstract

**Background and purpose:**

Since patients with primary brain tumor are expected to become long-term survivors, the prevention of long-term treatment-induced side effects is particularly important. This study aimed to explore whether symptom experience and symptom distress change over five years in adults with primary brain tumors treated with proton therapy. An additional aim was to explore whether symptom experience and symptom distress correlate.

**Materials and methods:**

The study had a longitudinal observational design. Adult (≥18 years) patients (n = 170) with primary brain tumors treated with proton therapy were followed over five years. Symptom experience and symptom distress were evaluated using the patient-reported Radiotherapy-Related Symptom Assessment Scale. Data from baseline, 1, 12, and 60 months were analyzed using non-parametric tests.

**Results:**

Of the 170 patients, the levels of symptoms and symptom distress were low. Fatigue increased at 1 (p=0.005) and 12 months (p=0.025) and was the most frequent symptom from baseline to 60 months’ follow-up. Cognitive impairment increased at 12 (p=0.027) and 60 months (p<0.001) and was the most distressing symptom at 60 months’ follow-up. There were significant, moderate to strong, correlations at all time points between symptom experience and symptom distress of fatigue, insomnia, pain, dyspnea, cognitive impairment, worry, anxiety, nausea, sadness, constipation, and skin reactions.

**Conclusion:**

Symptom experience and symptom distress changed in intensity over time with cognitive impairment as the most distressing symptom at 60 months. Future research should focus on identifying effective interventions aimed at alleviating these symptoms and reducing symptom distress for this vulnerable group of patients.

## Highlights

• Symptom experience and symptom distress changed in intensity over time.
• Cognitive impairment was the most distressing symptom at 60 months’ follow-up.
• Reduced levels of distressing symptoms would lead to a better HRQoL.

## Introduction

Globally, around 308,000 people are diagnosed annually with primary brain tumors, with over 1100 cases reported each year in Sweden, where approximately 60% are malignant. [1] With a five-year overall survival of 35%, malignant primary brain tumors remain among the most difficult cancers to treat. [2] The prognosis varies with the type of tumor, presence of isocitrate dehydrogenase (IDH) enzymes mutation and codeletion of 1p19q, how far it has spread at the time of diagnosis, general condition, age of the patient, and location in the brain. The enzyme codeletion of 1p19q is a pathognomic biomarker that defines the type of glioma and the potential prognosis. Codeletion of 1p19q and the presence of IDH mutation in an infiltrating or highly malignant glioma is associated with a significantly improved prognosis. [2], [3] Five-year survival for patients with glioblastoma is poor, 7%, while the prognosis for less aggressive forms is significantly better. [4] Further, an integrative genomic analysis, performed by the Cancer Genome Atlas Network, showed that patients with oligodendrogliomas (IDH-mutant, 1p/19q codeletion) had a better overall survival (OS) than those with astrocytomas. [5] The relative five-year survival in Sweden for men with a primary brain tumor is 50 % and the corresponding figure for women is 69%. [6]

Treatment for primary brain tumors (PBT) often includes a combination of surgery and radiotherapy (typically 60 Gy in 30–35 fractions) followed by six cycles of adjuvant chemotherapy. [7] In the Swedish national care program for tumors in the brain and spinal cord [8] it is recommended that proton therapy (PT) should be considered for all patients with grade 1–2 tumors, and patients with grade 3 tumors with a good prognosis factor (codeletion of 1p19q and/or IDH mutation).

PT is an emerging radiotherapy modality and is a beneficial treatment option for PBT [9], [10] and gives less risk of long-term side effects. [10]

Cancer survivors with PBT are at particular risk of long-term side effects because of the insult to the brain during cranial irradiation, surgical treatment, and the toxicity of chemotherapeutic agents. [11] In advanced cancer, symptoms seldom manifest in isolation, which emphasizes the importance of addressing symptoms in adults undergoing proton therapy for PBT. Patients with PBT often experience multiple concurrent symptoms over their disease trajectories. [12], [13], [14] The occurrence of multiple symptoms is associated with decreases in functional status and quality of life (QoL), and the experience of multiple symptoms is associated with higher levels of symptom distress. [12], [15] Symptom distress has been defined by McCorkle and Young [16] as “the degree of discomfort from the specific symptom being experienced as perceived by the patient”. Distress may be caused by physical, psychological, emotional, or social problems because of illness. [17] Patients with a primary brain tumor are likely to experience high levels of distress at some point during the disease trajectory [18], due to the unfavorable prognosis and the severe functional sequelae. [15] In a study by Randazzo et al. [17] distress-related symptoms were identified in PBT patients. Notably, symptoms such as fatigue, memory and concentration issues, sleep disturbance, and worry were associated with distress. Furthermore, higher levels of distress were reported to be correlated with lower health-related quality of life (HRQoL). Screening only for symptoms has been shown to be predictive of the most distressing symptoms but may not be completely accurate. A combination of assessment of symptoms as well as the patient's prioritization of the distress of symptoms can be of great help to health-care providers. Distress screening has many potential clinical benefits as facilitating communication, stimulating quality improvement in clinical care, provide clinically relevant information and can help health-care providers to develop interventions that may support quality of life. [19], [15]

The traditional metric used in oncology to demonstrate the goal in therapeutic studies is prolonged survival or prolonged time to disease progression. [20], [21] It has become increasingly important to involve patients in their own care, and to demonstrate that therapy improves patient function and HRQoL. This has contributed to an increasing interest in integrating patient-reported outcomes (PROs) into routine oncology. [20], [22]

Previous studies have shown that monitoring PROs was associated with higher patient satisfaction and improved symptom control. [23] According to Möllerberg et al. [24], there was poor agreement between PROs and clinician-reported outcomes skin reactions due to PBT. Kroeze et al [25] reported that physician reported comparable to patients in the direct post therapeutic phase, but not in the later follow-up. The poor agreement shows that the patient needs to be involved in the assessment of symptoms and their care.

Since patients with primary brain tumors are expected to become long-term survivors, the prevention of long-term treatment-induced side effects is particularly important. Further research, including long-term follow-up of treatment-induced side effects has thus become increasingly important in this patient group.[26], [27] Therefore, this study aimed to explore whether symptom experience and symptom distress change over five years in adults with PBT treated with PT. An additional aim was to explore whether symptom experience and symptom distress correlate. Findings from this study can form the basis for the development of effective interventions aimed at alleviating symptom experience, reducing patient symptom distress, and contributing to the rehabilitation of this vulnerable patient group.

## Materials and methods

This study is part of a larger multicenter research project (ProtonCare) in collaboration with the ProtonCare Study Group (PCSG) national research group.

### Study design

The study had a longitudinal observational design.

### Settings

The Skandion Clinic, the only proton clinic in Sweden, is situated in Uppsala. Eligible patients identified for PT or conventional radiotherapy (CRT) undergo comprehensive evaluation during a weekly national treatment video conference involving the Skandion Clinic and all seven university hospitals’ radiotherapy departments. All preparations, dose planning, and use of immobilization device are performed at the local radiotherapy department before the patients start their treatment at the Skandion Clinic. As the treatment is given as five fractions per week for 35 days, long-distance patients can stay at a patients’ hotel located in the same building. When completed PT, patients are re-referred to the home clinic for long-term follow-up.

### Participants

A consecutive sample of 171 patients diagnosed with a PBT treated with PT at the Skandion Clinic were asked to participate in the study.

Inclusion criteria were adult (≥18 years) patients diagnosed with a PBT, receiving PT, and able to communicate in Swedish. Patients diagnosed with a PBT grade 1–3 were included. Patients with a PBT grade 4 tumor were not included. Further inclusion criteria according to PRO-CNS are available in the registered protocol. [28] Exclusion criterion was an inability to understand and communicate in the Swedish language. Patients who did not comply with all inclusion criteria were excluded from obtaining valid results. Ethical approval was obtained from the Research Ethics Board in Gothenburg on 22 July 2015 (Dnr: 433-15).

### Data collection and procedures

Patients were included from August 2015 to May 2023. In this study we report data from baseline and 1, 12, and 60 months.

At the university hospitals in Sweden, a contact person was responsible for identifying eligible patients. A researcher (UL) from the PCSG asked the patients to participate and included them in the study. Study information was provided via telephone by the researcher. Written information about the study was sent by mail, together with a consent form. Based on the patient’s choice, data were collected either through a web interface or in paper format. The electronic questionnaires were available for retrieval from a database, and an e-mail was sent to the participant’s email address. Paper questionnaires were distributed to the participants by a radiation therapist in the Skandion Clinic, at baseline, and at all follow-ups by a researcher from PCSG. Participants received a prepaid envelope and were asked to return the questionnaires by mail. A reminder was sent to the postal address of participants who did not return the questionnaires within 10 days. Data regarding the tumor disease and treatment were collected from the participants’ medical records. Demographic data were obtained at baseline by a study-specific questionnaire.

### Questionnaires

The degree of symptoms and symptom distress were evaluated using the patient-reported questionnaire, the Radiotherapy-Related Symptom Assessment Scale (RSAS).[29] The questionnaire consists of the two dimensions experience and symptom distress. The RSAS has been evaluated against the established measuring instrument EORTC QLQ-C30 [30] and found to be a reliable, responsive, and valid questionnaire suitable for assessing symptom experience and symptom distress in PBT patients treated with PT. [29] RSAS assesses symptom intensity and symptom distress and comprises a core symptom set of 13 items; see Appendix 1. The questionnaire includes an assessment of symptom intensity from 1 (of no concern) to 4 (of greatest concern) and symptom distress associated with symptoms from 1 (of no concern) to 4 (of greatest concern). Each item score was converted to a continuous score ranging from 0 (not at all), to 33.3 (a little), 66.6 (quite a bit), and 100 (very much), with higher scores indicating more severe symptoms. [29]

### Statistical analysis

Mean, standard deviation, minimum, and maximum were used for age, while the number and percent were used for categorical variables. Descriptive statistics were used to summarize patients’ demographic and clinical characteristics and to analyze the intensity of participants’ daily symptoms. Before conducting the analyses, the data were initially examined by reviewing scatterplots, histograms, and Q-Q plots. The preliminary analyses showed that the score distribution for neither symptom nor symptom distress was normally distributed, and a non-parametric approach was adopted. The Wilcoxon signed-rank test was used for continuous variables to compare changes in symptom experience and symptom distress from baseline up to 60 months. Mann Whitney U-test was used to compare gender and age group differences. Spearman’s correlation coefficient was used to explore whether symptom experience and symptom distress correlate. Correlations <0.30 were considered low; correlations 0.30 to 0.60, moderate; and correlations >0.60 as strong. [31] A p-value of <0.05 was considered statistically significant. For interpretation purposes the most appropriate effect size measure, the rank-biserial correlation coefficient (r), was used. It provides an estimate of the strength and direction of the relationship between the independent groups and the outcome variable. The rank-biserial correlation coefficient ranges from -1 to 1, where -1 indicates a perfect negative relationship, 0 signifies no relationship, and 1 denotes a perfect positive relationship. [32] The analyses were focused on the baseline to 1-, 12-, and 60-month changes in symptom experience and symptom distress. The time points were chosen with the intention of including both acute and late effects. Time points were clinically relevant to compare the acute, subacute and late effects.

## Results

The sample comprised 170 patients; see Table 1. There were 98 (58%) men and 72 (42%) women, and the mean age was 45 years (range 19–77 years). Characteristics of the study population are shown in Table 2.

**Table 1:** Response rate

Time point	N	Percentage (%)
Baseline	170	99
1 month post treatment	63	37
12 months post treatment	73	43
60 months post treatment	54	32
A consecutive sample of 171 patients diagnosed with a malignant brain tumor treated with PT at the Skandion Clinic were asked to participate in the study. Patients were included from August 2015 to May 2023. In this study we report data from baseline and 1, 12, and 60 months’ follow-up. The sample comprised 170 patients.

**Table 2:** Baseline demographics and clinical characteristics

Characteristics	N
	n = 170
**Sex**	
Men, n (%)	98 (58)
Women, n (%)	72 (42)
**Age, years**	
Mean (SD)	45 (12)
Median (at 60 months)	43 (42,5)
Minimum	19
Maximum	77
**Civil status**	
Married, n (%)	122 (72)
Single, n (%)	48 (28)
**Education**	
Elementary, n (%)	13 (8)
Secondary, n (%)	62 (36)
University, n (%)	89 (52)
Missing, n (%)	6 (4)
**Diagnosis (min/max, mean age)**	
C31.8: Malignant neoplasm of overlapping sites of accessory sinuses (61/61,61)	1
C70.0: Malignant neoplasm of cerebral meninges (28/68,51)	7
C71.1: Malignant neoplasm of frontal lobe (26/57,42)	33
C71.2: Malignant neoplasm of temporal lobe (25/53,36)	7
C71.3: Malignant neoplasm of parietal lobe (34/45, 37)	5
C71.4: Malignant neoplasm of occipital lobe (31/31,31)	1
C71.5: Malignant neoplasm of cerebral ventricle (25/58,46)	4
C71.6: Malignant neoplasm of cerebellum (30/53,42)	2
C71.7: Malignant neoplasm of brain stem (70/70,70)	1
C71.8: Malignant neoplasm of overlapping sites of brain (32/40,36)	2
C71.9: Malignant neoplasm of brain, unspecified (19-69,43)	87
C72.0: Malignant neoplasm of spinal cord (23/65,44)	2
C75.1: Malignant neoplasm of pituitary gland (28/28,28)	1
C79.3: Secondary malignant neoplasm (27/48,39)	4
C90.3: Plasmacytoma, solitary (57/57,57)	1
Missing (38–77,56)	12

The results demonstrated that symptom experience and symptom distress changed over time. Participants reported low levels (0–33.3) of symptom experience except for fatigue at 12-months follow up (>33.3). Further, participants reported low levels (0–33.3) of symptom distress throughout the study. The results demonstrated that a correlation exists between symptom experience and symptom distress. Changes over time in experience of symptoms followed the same pattern as symptom distress. Higher levels of symptom experience, i.e. more severe symptoms, were correlated with higher levels of symptom distress, and vice versa.

### Symptom changes over time

The assessment of changes of symptom experience from baseline to various time points post-treatment revealed the following: from baseline to 1-month post-treatment, symptoms of fatigue (p = 0.05, r = −0.49), loss of appetite (p = 0.003, r = −0.69), cognitive impairment (p = 0.023, r = −0.56), nausea (p = 0.011, r = −0.65), and skin reactions (p < 0.001, r = −1.00) increased significantly. Further there was a significant increase in fatigue (p = 0.023, r = −0.43), pain (p = 0.003, r = −0.50), cognitive impairment (p < 0.001, r = −0.71), constipation (p = 0.005, r = −0.80), and skin reactions (p = 0.006, r = −0.86) from baseline to 12-month follow-up. At 60 months’ follow-up loss of appetite (p = 0.025, r = –0.76), cognitive impairment (p = 0.002, r = –0.42), and skin reactions (p = 0.037, r = –1.0) increased significantly compared to baseline. Symptoms of worry had the highest levels at baseline and decreased at each time point up to 60 months of follow-up. See Table 3.

**Table 3:** Symptom change, from baseline up to 1-, 12- and 60-month**s** follow-up

Symptom	Base line (N=170,99%)	1 month post treatment (N=63, 37%)	12 months post treatment (N=73, 43%)	60 months post treatment (N=54, 32%)	Change from baseline to 1 month	P-value/r	Change from baseline to 12 months	P-value/r	Change from baseline to 60 months	P-value/r
Fatigue	27.5 (28.6) 33.3 (0.0-100.0)	43.2 (32.4) 33.3 (0.0–100.0)	33.8 (32.2) 33.3 (0.0–100.0)	31.5 (30.0) 33.3 (0.0–100.0)	15.6 (36.1)	**0.005/−0.49**	9.6 (31.2)	**0.025/−0.43**	7.4 (35.3)	0.200/−0.25
Insomnia	23.1 (29.5) 0.0 (0.0-100.0)	21.2 (30.1) 0.0 (0.0–100.0)	21.9 (31.5) 0.0 (0.0–100.0)	21.0 (26.1) 0.0 (0.0–100.0)	0.0 (32.2)	1.000/0.00	2.3 (29.0)	0.403/−0.17	−2.5 (36.5)	0.655/0.10
Pain	11.0 (20.1) 0.0 (0.0-66.7)	16.4 (23.1) 0.0 (0.0–100.0)	15.5 (25.5) 0.0 (0.0–100.0)	13.6 (23.8) 0.0 (0.0–66.7)	4.2 (20.3)	0.106/−0.40	5.5 (22.2)	**0.030/−0.50**	4.9 (30.0)	0.162/−0.32
Loss of appetite	5.3 (15.1) 0.0 (0.0-100.0)	15.3 (24.6) 0.0 (0.0–100.0)	5.9 (16.0) 0.0 (0.0–66.7)	9.3 (13.7) 0.0 (0.0–100.0)	9.5 (24.3)	**0.003/−0.69**	1.8 (16.6)	0.356/−0.29	8.0 (25.0)	**0.025/−0.76**
Dyspnea	1.0 (5.65) 0.0 (0.0-33.3)	2.7 (9.1) 0.0 (0.0–33.3)	3.2 (9.9) 0.0 (0.0–33.3)	2.5 (8.8) 0.0 (0.0–33.3)	1.1 (8.4)	0.424/−0.50	2.3 (10.1)	0.073/−0.71	1.9 (10.1)	0.233/−0.60
Cognitive impair-ment	14.7 (22.0) 0.0 (0.0–100.0)	24.3 (26.9) 33.3 (0.0–100.0)	23.7 (28.6) 33.3 (0.0–100.0)	22.8 (26.6) 16.7 (0.0–100.0)	7.4 (24.3)	**0.023/−0.56**	11.0 (23.6)	**<0.001/−0.71**	11.7 (26.0)	**0.002/−0.42**
Worry	26.1 (26.0) 33.3 (0.0–100.0)	19.0 (26.6) 0.0 (0.0–100.0)	18.3 (26.7) 0.0 (0.0–100.0)	16.0 (24.0) 0.0 (0.0–100.0)	−5.8 (20.3)	0.059/0.46	−3.2 (29.0)	0.502/0.12	−10.50 (36.5)	0.060/0.39
Anxiety	11.0 (21.4) 0.0 (0.0–100.0)	8.5 (19.8) 0.0 (0.0–100.0)	9.1 (19.5) 0.0 (0.0–66.7)	8.0 (17.1) 0.0 (0.0–66.7)	-1.6 (16.3)	**0.464/**0.23	0.5 (19.6)	**0.843/−**0.05	−2.5 (23.2)	0.447/0.20
Nausea	4.2 (13.2) 0.0 (0.0–100.0)	12.2 (23.4) 0.0 (0.0–100.0)	5.5 (14.7) 0.0 (0.0–66.7)	3.7 (12.4) 0.0 (0.0–66.7)	7.9 (23.7)	**0.011/−0.65**	2.7 (14.4)	0.120/−0.50	1.9 (13.6)	0.376/−0.43
Sadness	15.7 (22.4) 0.0 (0.0–100.0)	16.4 (25.3) 0.0 (0.0–100.0)	15.5 (23.6) 0.0 (0.0–66.7)	13.0 (23.7) 0.0 (0.0–100.0)	0.5 (20.3)	0.708/−0.08	1.8 (23.5)	0.507/−0.13	−3.1 (28.4)	0.499/0.14
Constipa-tion	3.3 (11.3) 0.0 (0.0–66.7)	9.0 (20.9) 0.0 (0.0–100.0)	10.5 (24.1) 0.0 (0.0–100.0)	8.6 (18.5) 0.0 (0.0–66.7)	5.8 (23.6)	0.064/−0.51	6.6 (20.0)	**0.005/−0.80**	4.3 (21.5)	0.143/−0.43
Diarrhea	6.1 (17.3) 0.0 (0.0–100.0)	7.4 (16.3) 0.0 (0.0–66.7)	2.3 (12.8) 0.0 (0.0–100.0)	3.7 (12.4) 0.0 (0.0–66.7)	2.1 (15.7)	0.308/−0.33	−2.7 (17.4)	0.212/0.47	−1.85e−5 (14.5)	1.000/0.00
Skin reactions	2.7 (9.0) 0.0 (0.0–66.7)	13.2 (19.4) 0.0 (0.0–66.7)	7.8 (17.1) 0.0 (0.0–66.7)	4.3 (11.3) 0.0 (0.0-33.3)	11.1 (18.0)	**<0.001/−1.00**	5.5 (15.7)	**0.006/−0.86**	3.1 (9.8)	**0.037/−1.00**
The table show means (standard deviations)/medians (min, max)/n. For comparisons over time, the Wilcoxon signed rank test was used. High symptom scores indicate more severe symptoms. Statistical significance was assumed at the p < 0.05 level. Rank biserial correlation (r) was used as effect size estimate. Bold style/red highlighted indicates significant result.										

### Symptom distress changes over time

All symptoms of distress changed over the five years. Distress of fatigue increased significantly from baseline to 1 month (p = 0.017, r = −0.49) and from baseline to 12 months’ follow up (p = 0.015, r = −0.57). Distress of cognitive impairment increased significantly from baseline to 1 month (p = 0.027, r = −0.60) and from baseline to 12 months’ follow up (p < o.001, r = −0.92). Distress of constipation increased significantly (p = 0.31, r = −1.00). Cognitive impairment increased successively at all time points from baseline and was the most distressing symptom at 60-month follow-up. See Table 4.

**Table 4:** Distress change, from baseline up to 1-, 12- and 60-month follow up

Symptoms of distress	Baseline (N=170, 99%)	1 month post treatment (N=63, 37%)	12 months post treatment (N=73, 43%)	60 months post treatment (54, 32%)	Change from baseline to 1 month	P-value/r	Change from baseline to 12 months	P-value/r	Change from baseline to 60 months	P-value/r
Fatigue	9.8 (21.3) 0.0 (0.0–100.0)	22.2 (29.3) 0.0 (0.0–100.0)	16.4 (27.3) 0.0 (0.0–100.0)	12.3 (20.8) 0.0 (0.0–66.7)	11.6 (34.5)	**0.017/−0.49**	8.7 (26.7)	**0.015/−0.57**	4.9 (25.4)	0.190/−0.35
Insomnia	10.6 (23.6) 0.0 (0.0–100.0)	9.5 (20.2) 0.0 (0.0–66.7)	12.3 (25.8) 0.0 (0.0–100.0)	9.3 (20.9) 0.0 (0.0–100.0)	1.1 (26.8)	0.732/−0.10	−0.7 (22.6)	0.151/−0.38	−3.1 (26.1)	0.391/0.23
Pain	4.9 (14.8) 0.0 (0.0-66.7)	7.4 (19.3) 0.0 (0.0-100.0)	8.7 (22.9) 0.0 (0.0-100.0)	6.8 (18.7) 0.0 (0.0-66.7)	3.2 (18.7)	0.212/−0.47	4.6 (25.6)	0.096/−0.51	2.5 (23.2)	0.385/−0.30
Loss of appetite	1.2 (8.8) 0.0 (0.0–100.0)	4.2 (14.0) 0.0 (0.0–66.7)	1.4 (8.7) 0.0 (0.0–66.7)	0.6 (4.5) 0.0 (0.0–33.3)	3.2 (13.0)	0.071/−0.75	5.0 (23.4)	0.371/−1.00	0.0 (0.0)	1.000/−1.00
Dyspnea	0.2 (2.6) 0.0 (0.0–33.3)	1.1 (5.9) 0.0 (0.0–33.3)	2.3 (10.1) 0.0 (0.0–66.7)	0.6 (4.5) 0.0 (0.0–33.3)	0.0 (0.0)	0.346/−1.00	0.0 (0.0)	0.089/−1.00	0.0 (0.0)	1.000/−1.00
Cognitive impairment	5.7 (16.6) 0.0 (0.0–100.0)	10.1 (21.3) 0.0 (0.0–100.0)	11.9 (23.8) 0.0 (0.0–100.0)	14.8 (23.0) 0.0 (0.0–66.7)	2.7 (23.4)	0.367/−0.32	7.3 (23.7)	**0.027/−0.60**	13.0 (21.9)	**<0.001/−0.92**
Worry	10.2 (20.8) 0.0 (0.0–100.0)	7.9 (23.0) 0.0 (0.0–100.0)	8.7 (20.1) 0.0 (0.0–66.7)	7.4 (21.1) 0.0 (0.0–100.0)	−2.1 (15.7)	0.351/0.39	2.3 (17.9)	0.303/−0.33	−4.3 (29.0)	0.378/0.23
Anxiety	5.7 (16.2) 0.0 (0.0–100.0)	5.8 (19.4) 0.0 (0.0–100.0)	3.7 (10.5) 0.0 (0.0–33.3)	3.7 (12.4) 0.0 (0.0–66.7)	−0.5 (15.3)	0.860/0.11	−0.9 (14.7)	0.627/0.17	−1.2 (15.8)	0.608/0.20
Nausea	1.4 (9.1) 0.0 (0.0–100.0)	4.2 (14.0) 0.0 (0.0–66.7)	0.9 (5.5) 0.0 (0.0–33.3)	0.6 (4.5) 0.0 (0.0–33.3)	2.7 (15.0)	0.187/−0.57	0.0 (7.9)	1.000/0.00	0.0 (0.0)	1.000/−1.00
Sadness	5.3 (16.8) 0.0 (0. –100.0)	6.9 (20.0) 0.0 (0.0–100.0)	4.6 (14.0) 0.0 (0.0–66.7)	8.0 (21.4) 0.0 (0.0–100.0)	0.5 (16.4)	0.903/−0.07	2.7 (15.5)	0.145/−0.51	5.6 (26.5)	0.133/−0.55
Constipation	1.4 (8.4) 0.0 (0.0–66.7)	3.2 (13.0) 0.0 (0.0–66.7)	6.4 (20.5) 0.0 (0.0–100.0)	1.2 (6.4) 0.0 (0.0–33.3)	1.1 (15.8)	0.679/−0.27	4.1 (15.7)	**0.031/−1.00**	0.0 (11.2)	1.000/0.00
Diarrhea	1.4 (9.1) 0.0 (0.0–100.0)	1.6 (9.3) 0.0 (0.0–66.7)	0.9 (7.8) 0.0 (0.0–66.7)	0.0 (0.0) (0.0–0.0)	1.1 (10.3)	0.586/−0.50	0.5 (8.8)	1.000/−0.33	0.0 (0.0)	1.000/1.00
Skin reactions	0.6 (5.7) 0.0 (0.0–66.7)	1.6 (7.2) 0.0 (0.0–33.3)	2.3 (10.1) 0.0 (0.0–66.7)	0.6 (4.5) 0.0 (0.0–33.3)	0.5 (7.3)	0.773/−0.33	1.4 (6.7)	0.149/−1.00	0.0 (0.0)	1.000/−1.00
The table show means (standard deviations)/medians (min, max)/n. For comparisons over time, the Wilcoxon signed rank test was used. High symptom scores indicate more severe symptoms. Statistical significance was assumed at the p < 0.05 level. Rank biseral correlation (r) was used as effect size estimate. Bold style/red highlighted indicates significant result.										

### Symptom and distress

**Graph 1:**
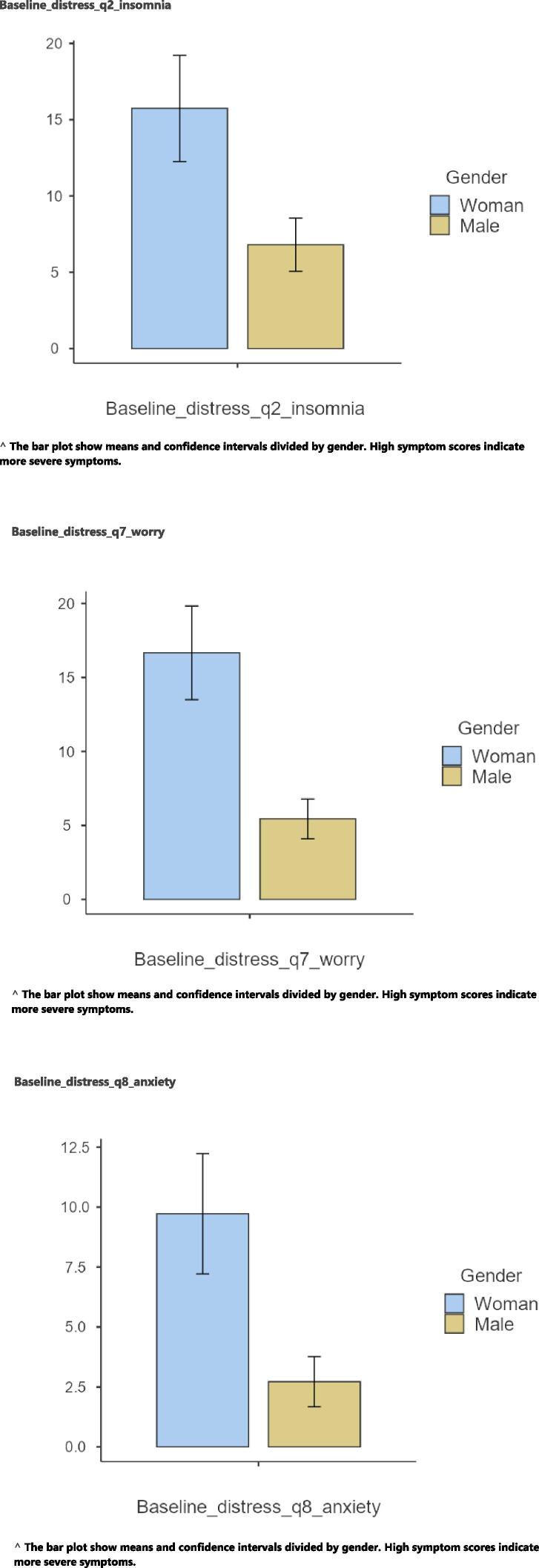
Baseline distress of symptoms. Baseline_distress_q2_insomnia.^The bar plot show means and confidence intervals divided by gender. High symptom scores indicate more severe symptoms.

At baseline, women reported higher levels of all symptoms compared to men except for dyspnea and diarrhea. Women reported significantly higher levels of symptoms of fatigue (p = 0.009, r = 0.22), anxiety (p = 0.002, r = 0.21), sadness (p 0.016, r 0.19), and constipation (p = 0.012, r = 0.11) compared to men. Levels of distress of insomnia (p = 0.049, r = 0.12), worry (p = 0.002, r = 0.21), and anxiety (p = 0.008, r = 0.14) were at significantly higher levels for women than men. There were no significant differences in symptom experience and/or symptom distress between the age groups (Graph 1).

**Graph 2:**
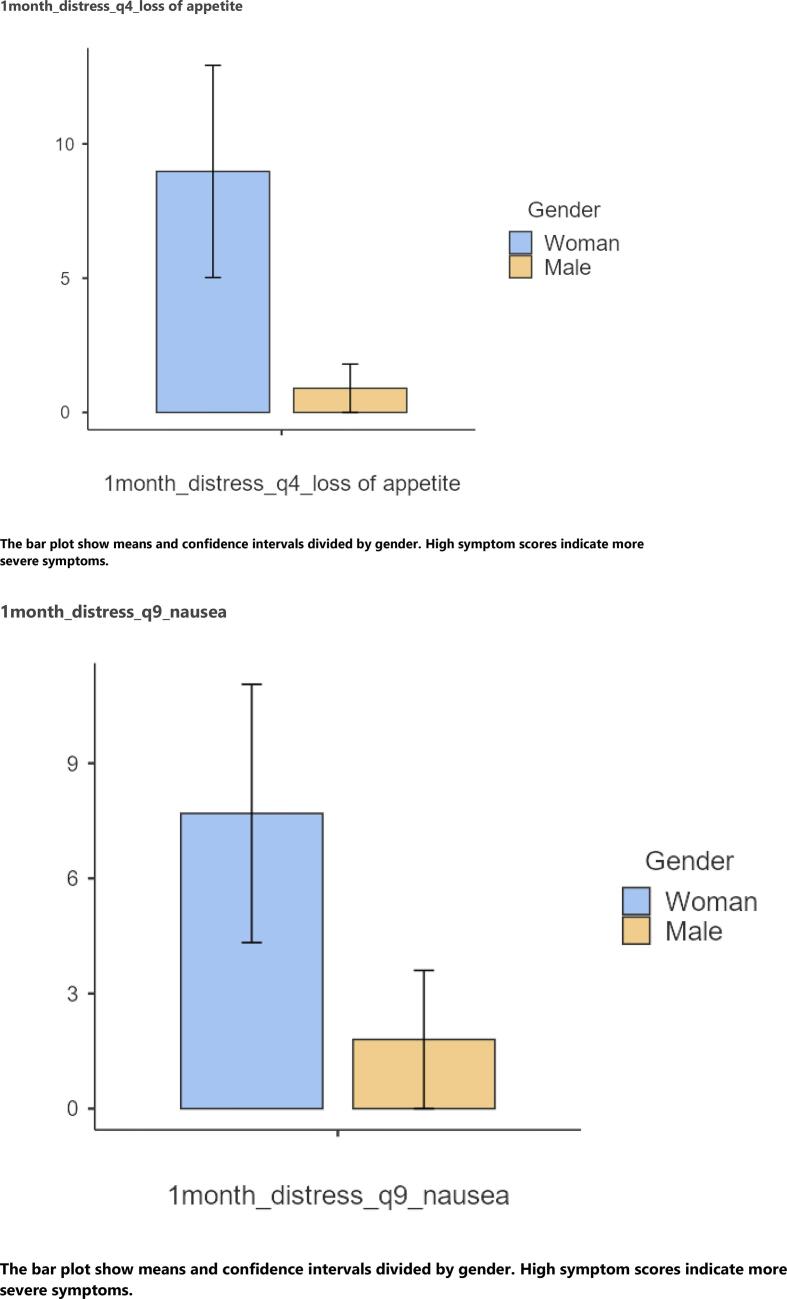
1-month follow-up distress of symptoms. 1month_distress_q4_loss of appetite. The bar plot show means and confidence intervals divided by gender. High symptom scores indicate more severe symptoms.

At 1-month follow-up, women reported significantly higher levels of nausea (p = 0.045, r = 0.23) than men. Further, women reported significantly higher levels of distress of loss of appetite (p = 0.028, r = 0.17) and nausea (p = 0.035, r = 0.17). No significant differences in symptom experience and/or symptom distress were found between the age groups (Graph 2).

**Graph 3:**
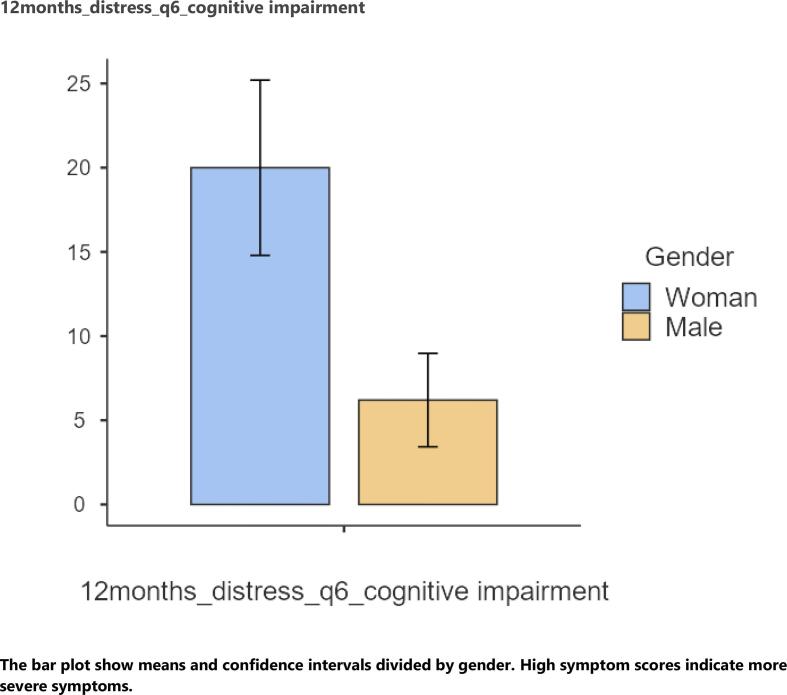
12-months follow-up distress of symptoms. 12months_distress_q6_cognitive impairment. The bar plot show means and confidence intervals divided by gender. High symptom scores indicate more severe symptoms.

At 12-month follow-up, women reported significantly higher symptoms for dyspnea (p = 0.013, r = 0.18) and sadness (p = 0.008, r = 0.31) than men. Women reported significantly higher levels of distress of cognitive impairment (p = 0.032, r = 0.24) compared to men. Patients aged >43 years reported significantly higher appetite loss symptoms (p = 0.013, r = 0.20) than patients aged <43 years. Further, patients >43 years reported significantly higher levels of distress of sadness (p = 0.041, r = 0.15) than patients aged <43 years (Graph 3).

At the 60-month follow-up, no significant differences existed between men and women in reported symptoms or symptom distress. Patients aged <43 years reported significantly higher symptoms of worry (p = 0.022, r = 0.31) than patients >43 years. No significant differences were found between the age groups in distress of symptoms.

### Correlation between symptom and symptom distress

Overall, for all time points, the result demonstrated a significant (p < 0.05) correlation between symptom experience and symptom distress, except for dyspnea, at baseline, and diarrhea at the 60-month follow-up. Notably, symptoms of skin reaction changes were significant but were less related to distress than cognitive impairment. (Table 5).

**Table 5:** Correlation matrix, Spearman’s correlation, symptom and distress

Variable	Baseline	1 month post treatment	12 months post treatment	60 months post treatment
Fatigue	0.635/<0.001	**0.778/<0.001**	**0.806/<0.001**	0.684/<0.001
Insomnia	0.667/<0.001	0.702/<0.001	0.754/<0.001	0.671/<0.001
Pain	0.622/<0.001	0.630/<0.001	0.718/<0.001	0.676/<0.001
Loss of appetite	0.423/<0.001	0.541/<0.001	0.467/<0.001	0.353/0.009
Dyspnea	−0.013/0.862	0.617/<0.001	0.739/<0.001	0.486/<0.001
Cognitive impairment	0.589/<0.001	0.658/<0.001	0.709/<0.001	**0.782/<0.001**
Worry	0.600/<0.001	0.604/<0.001	0.675/<0.001	0.633/<0.001
Anxiety	**0.726/<0.001**	0.705/<0.001	0.715/<0.001	0.666/<0.001
Nausea	0.523/<0.001	0.608/<0.001	0.441/<0.001	0.421/0.002
Sadness	0.523/<0.001	0.635/<0.001	0.604/<0.001	0.738/<0.001
Constipation	0.574/<0.001	0.590/<0.001	0.722/<0.001	0.408/0.002
Diarrhea	0.447/<0.001	0.445/<0.001	0.585/<0.001	NaN
Skin reactions	0.447/<0.001	0.442/<0.001	0.555/<0.001	0.356/0.008
The table shows Spearman’s rank correlation (rho) between between symptoms and distress. Correlations <0.30 were considered low; correlations 0.30 to 0.60, moderate; and correlations >0.60, strong. Statistical significance was assumed at the p < 0.05 level. Bold style/red highlighted indicates the highest correlation per time point.				

At baseline, a positive moderate-to-strong correlation (Spearman’s rho 0.42–0.73, p < 0.001) was found for all symptoms, except for dyspnea. The strongest correlation between symptom and symptom distress was found for anxiety (Spearman’s rho 0.73, p-value <0.001).

At 1-month follow-up there was a moderate-to-strong correlation (Spearman’s rho 0.44–0.78, p < 0.001) for all symptoms. The strongest correlation was found for fatigue (Spearman’s rho 0.78, p < 0.001).

A moderate-to-strong positive correlation (Spearman’s rho 0.44–0.81, p < 0.001) was found for all symptoms at 12-month follow-up with the strongest correlation between symptom experience and distress of fatigue (Spearman’s rho 0.81, p < 0.001).

At the 60-month follow-up there was a moderate-to-strong correlation (Spearman’s rho 0.36–0.78, p < 0.001–0.009) for all symptoms except for diarrhea. At 60-month follow-up, the strongest correlation was found for cognitive impairment (Spearman’s rho 0.78, p < 0.001).

## Discussion

This longitudinal observational study demonstrated that symptom experience and symptom distress were low (0-33.3) for patients with malignant brain tumor before and up to 5 years after receiving PT. Experience of fatigue, the most frequent symptom at all time points, reached peak levels at 1 month but then returned to nearly the same level as baseline at 60 months.

At 60 months loss of appetite, cognitive impairment and skin reactions were still at significant higher levels compared to baseline. As Alemany et al. [11] found, cancer survivors with PBT are at particular risk of long-term side effects because of the treatment regimes, surgery, radiation and chemotherapy. Tumor location and other concurrent symptoms also affect the patient's experience of symptoms.

In this study a large number of patients had frontal lobe tumors (n=33). This may have had an impact of the result regarding cognitive impairment. The frontal lobe plays a crucial role in advanced cognitive functions. Patients with malignant brain tumor experience cognitive impairment due to the location of the tumor, but also as a side effect of treatments such as radiation.[33] Taphoorn et al.[34] and Mayo et al.[35] found that patients with frontal tumors, underreport cognitive dysfunction due to impaired judgment.

Cognitive impairment is an obstacle to care and reintegration into society.[36] Both cognitive rehabilitation and pharmacological interventions have been used to treat cognitive impairment in patients with PBT. [37] Rehabilitative measures and interventions should be started already at diagnosis. Care and rehabilitation involves the multidisciplinary team throughout the disease trajectory. The diagnosis and subsequent treatment of malignant brain tumor can involve a lot of information for patients and caregivers to process. Thakkar et al. [37], suggest that special attention should be paid to communication challenges, family education and potential hospice referrals. There are several factors to consider when offering care, with the overall quality of life in mind. Caregivers should address common symptoms of patients with malignant brain tumors including side effects from chemotherapy, surgery and radiotherapy, which this study helps to draw attention to by highlighting the two dimensions of symptoms and symptom distress during a 5-year period after completion of PT. Many of these symptoms can be alleviated with the use of medications. But together, the multidisciplinary team can work to achieve the patient's goals and enrich their quality of life. This can be achieved through adapted rehabilitative measures and interventions through the patient's disease trajectory from first symptoms, with a particular focus on cognitive impairment that has proven to be an obstacle to readjustment to everyday life.

Jzerman-Korevaar et al. [13], found that patients with malignant brain tumors often experience multiple concurrent symptoms over their disease trajectories. The current study showed that patients experienced multiple concurrent symptoms and symptom distress that changed in intensity over time. Additionally, previous research showed that multiple symptoms are also associated with higher levels of symptom distress and decreased functional status and HRQoL. [12], [15]

In this study patients reported symptom distress caused by physical, psychological, and emotional problems, aligning with findings by Randazzo et al. [17] Further, this study showed that cognitive impairment increased successively from baseline and was the most distressing symptom at 60-month follow-up. Ek et al [38] found that patients with malignant brain tumors may have a stable cognitive function from the start, but cognitive impairment appeared during the seven years of follow-up. Otto-Meyer et al.’s [18] results indicate that patients with a primary brain tumor are likely to experience high levels of symptom distress at some point during the disease, which the results of this study could not verify.

The results demonstrated a significant, moderate-to-strong correlation at all time points between symptom experience and distress of fatigue, insomnia, pain, dyspnea, cognitive impairment, worry, anxiety, nausea, sadness, constipation, and skin reactions. Randazzo et al. [17] reported fatigue, memory and concentration issues, sleep disturbance, and worry associated with symptom distress, which accords with results of this study. Further, Randazzo et al. [17] reported that higher levels of symptom distress were associated with lower HRQoL. This emphasizes the importance of screening for symptom experience and symptom distress, at PT clinics and during long-term follow-up at home clinics, to detect and take measures that could reduce levels and lead to higher HRQoL.

PRO is considered a good basis for nursing patients with malignant brain tumors. Introducing PRO in clinical practice may lead to less symptom distress and higher symptom control levels than in patients receiving only standard care. Findings from Möllerberg et al. [24], and Kroeze et al. [25] emphasize that the patient needs to be involved in assessing symptoms and their care. This approach advocated by Basch et al. [23] could lead to higher patient satisfaction and better symptom control. As Armstrong et al. [20] state, patients want to live longer, but they also want to continue to function as well as possible for as long as possible. Moreover, screening for patients’ experience of symptom distress can further help healthcare providers develop interventions that may support QoL. [15]

The study’s main strength is that all data are patient-reported, offering patients’ perspectives of symptoms and symptom distress over a five-year period. Few studies to date have followed this population with these variables and methods. A further strength is that all patients receiving PT were invited to participate in the study.

Further, the RSAS lacks specificity tailored to patients with brain tumors, potentially impacting the precision of the obtained data. The RSAS is designed as a generic questionnaire for patients receiving radiotherapy and not as a diagnosis-specific questionnaire for patients with brain tumor.[29] Nevertheless, the RSAS is clinically relevant as the questionnaire responds to both dimensions, symptom experience and symptom distress which gives us guidance on what to focus on in our interventions.

Further, missing data is a major challenge and patients who have multiple symptoms and suffer from cognitive impairment are often the ones that may find it most difficult to complete questionnaires. A questionnaire that has minimal item burden and still capture the needed data are crucial for not missing data. In future RSAS could be modified to be better adapted to patients with malignant brain tumors.

A limitation is that the follow-up began after 2 years, which contributed to few patients reaching the 5-year follow-up when this study was conducted. This may have had an impact of the result and reduces the generalizability.

Thurin et al. [27] found that radiation doses to uninvolved contralateral structures can be reduced with PT, to adults with low-grade gliomas, compared to CRT. Further, they found sparse data on long-term treatment outcomes, but the results indicated that PT is comparable to CRT.

Medications commonly given to patients with PBT include seizure medications and steroids. Concurrent medication use may confound the study of symptoms. Both steroids and seizure medications can affect physical, emotional, and cognitive functioning. [39] It is a limitation of this study that we do not have access to that data for the entire population.

In future studies, it would be of great interest to follow the ability to work, social life, and relationships during these five years, reflecting another dimension of the QoL of patients with malignant brain tumors treated with PT. Further, a comparative analysis with a group undergoing CRT would provide valuable additional data/information.

## Conclusion

In conclusion, our study demonstrates that patients with malignant brain tumors undergoing proton therapy experience low levels of symptoms and symptom distress, both pre-treatment and up to 5 years post-treatment. The intensity of symptoms and symptom distress varies over time, with fatigue consistently emerging as a prominent symptom at all time points, and cognitive impairment becoming the most distressing symptom at the 60-month follow-up.

These findings underscore the interplay between symptom experience and symptom distress in this patient population. Therefore, future research must focus on identifying effective interventions aimed at alleviating these symptoms and reducing patient symptom distress. Such interventions have the potential to significantly enhance the QoL for this vulnerable group of patients.

## Informed Patient Consent

The author(s) confirm that written informed consent has been obtained from the involved patient(s) or if appropriate from the parent, guardian, power of attorney of the involved patient(s); and, they have given approval for this information to be published in this case report (series).

## Declaration of competing interest

The authors declare that they have no known competing financial interests or personal relationships that could have appeared to influence the work reported in this paper.
