# Supplementary material for: Symptom experience and symptom distress in patients with malignant brain tumor treated with proton therapy: A five-year follow-up study
Source: Tech Innov Patient Support Radiat Oncol. 2024 Aug 16;31:100269. doi: 10.1016/j.tipsro.2024.100269 (PMC11402412; doi:10.1016/j.tipsro.2024.100269)
Supplement: Supplementary Data 1 [file mmc1.docx]

| **We are interested in some things about you and your health. Please answer all of the questions yourself by circling the number of each symptom listed below that applies to you. Please also check the corresponding boxes for A (symptom intensity) and B (distress) that best reflect how you have experienced that symptom.** | | **A**    EXPERIENCE OF SYMPTOM INTENSITY | | | | **B**  DISTRESS | | | |
| --- | --- | --- | --- | --- | --- | --- | --- | --- | --- |
| **During the past 24 hours I have experienced…** | | Not at all | A little | Quite a bit | Very much | Of little or no distress | Of some distress | Of high distress | Of the highest distress |
|  | Fatigue | □ | □ | □ | □ | ○ | ○ | ○ | ○ |
|  | Insomnia | □ | □ | □ | □ | ○ | ○ | ○ | ○ |
|  | Pain | □ | □ | □ | □ | ○ | ○ | ○ | ○ |
|  | Loss of appetite | □ | □ | □ | □ | ○ | ○ | ○ | ○ |
|  | Dyspnea | □ | □ | □ | □ | ○ | ○ | ○ | ○ |
|  | Cognitive impairment | □ | □ | □ | □ | ○ | ○ | ○ | ○ |
|  | Worry | □ | □ | □ | □ | ○ | ○ | ○ | ○ |
|  | Anxiety | □ | □ | □ | □ | ○ | ○ | ○ | ○ |
|  | Nausea | □ | □ | □ | □ | ○ | ○ | ○ | ○ |
|  | Sadness | □ | □ | □ | □ | ○ | ○ | ○ | ○ |
|  | Constipation | □ | □ | □ | □ | ○ | ○ | ○ | ○ |
|  | Diarrhea | □ | □ | □ | □ | ○ | ○ | ○ | ○ |
|  | Skin reactions | □ | □ | □ | □ | ○ | ○ | ○ | ○ |
